# Supplementary material for: Frailty Syndrome as a Transition from Compensation to Decompensation: Application to the Biomechanical Regulation of Gait
Source: Int J Environ Res Public Health. 2023 May 30;20(11):5995. doi: 10.3390/ijerph20115995 (PMC10253052; doi:10.3390/ijerph20115995)
Supplement: Supplementary file 1 [file ijerph-20-05995-s001.zip › ijerph-2095577-supplementary.pdf]

Supplemental information

10 second fragments of raw triaxial accelerometry for a representative case of each study group

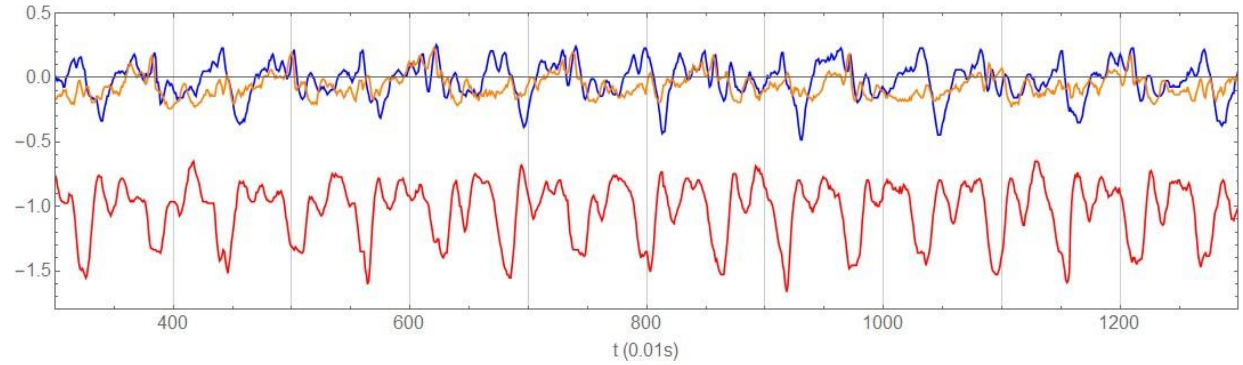

(a) Triaxial accelerometry of a selected control subject from C1 group

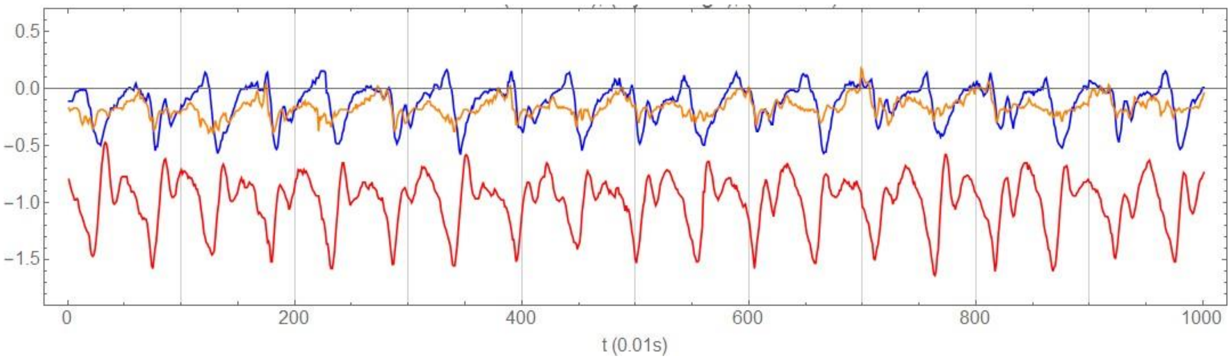

(b) Triaxial accelerometry of a selected control subject from C2 group

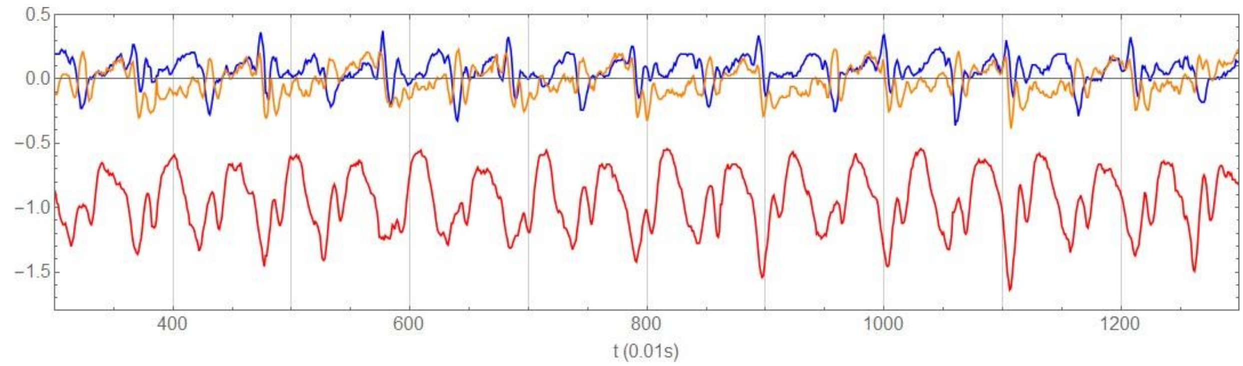

(c) Triaxial accelerometry of a selected non-frail older subject (nF)

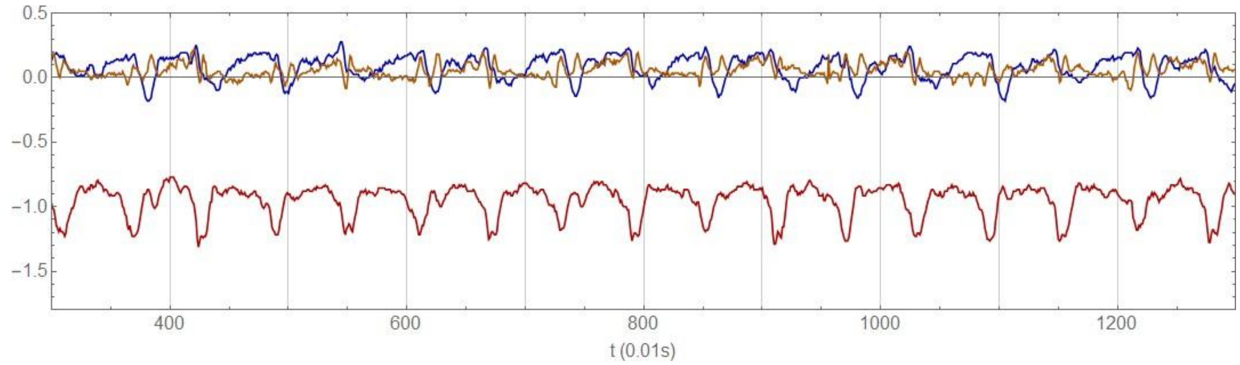

(d) Triaxial accelerometry of a selected frail older subject (F)

Figure S1. Fragments of 10 seconds of triaxial accelerometry at the beginning of a 160m walk of (a) a selected female young control subject of 24 yo, (b) a selected male control of 37 yo, (c) a selected female non-frail older adult of 74 yo, and (d) a selected male frail older adult of 81 yo. Every series represents acceleration in one direction of movement: anteroposterior a_{AP} (blue curves), mediolateral a_{ML} (orange curves) and vertical a_{VT} (red curves). 1s time intervals have been indicated with vertical gridlines. These series were measured with the triaxial accelerometer of the Zephyr Bioharness 3.0. The asymmetric location of the accelerometer below the left armpit allows to distinguish between left steps (larger amplitude) and right steps (smaller amplitude), see e.g., vertical acceleration a_{VT} for the selected young control adult.

Autocorrelation functions for the time series shown in Figure S1

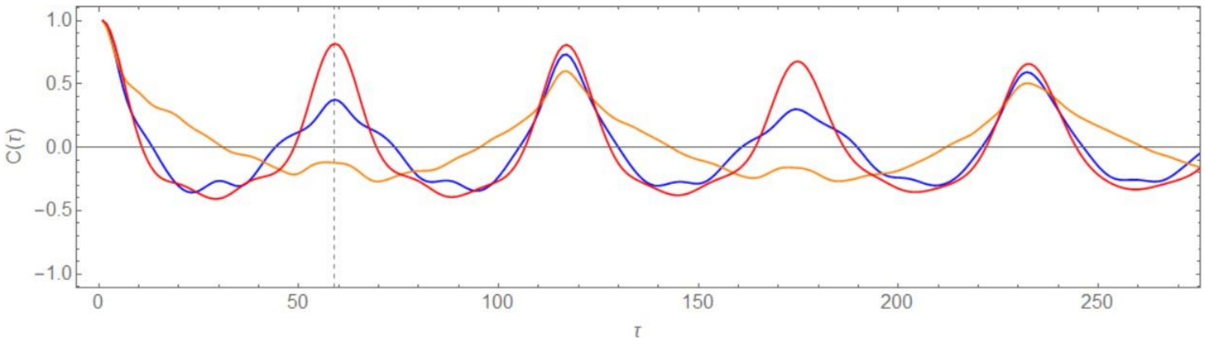

(a) Autocorrelation function of a selected control subject from C1 group

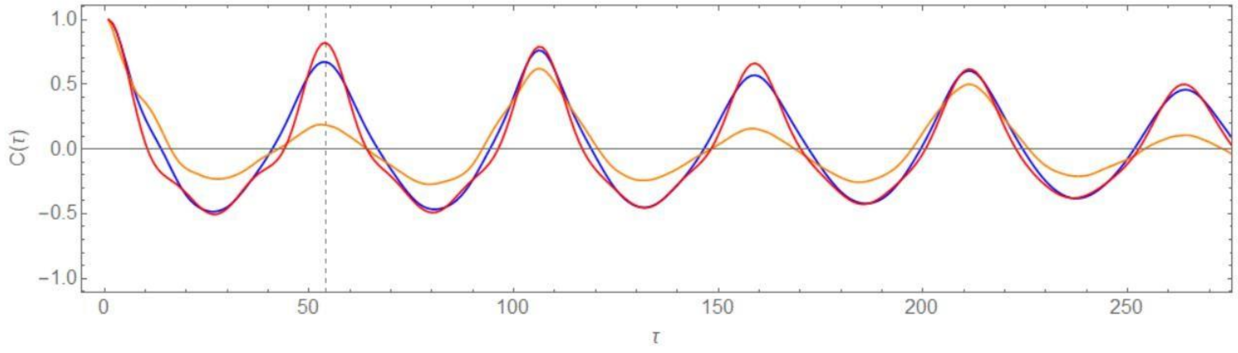

(b) Autocorrelation function of a selected control subject from C2 group

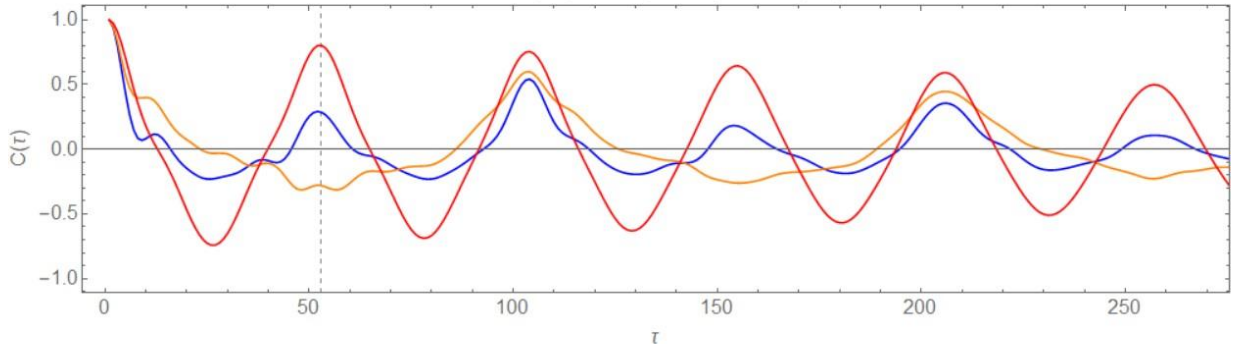

(c) Autocorrelation function of a selected non-frail older adult (nF)

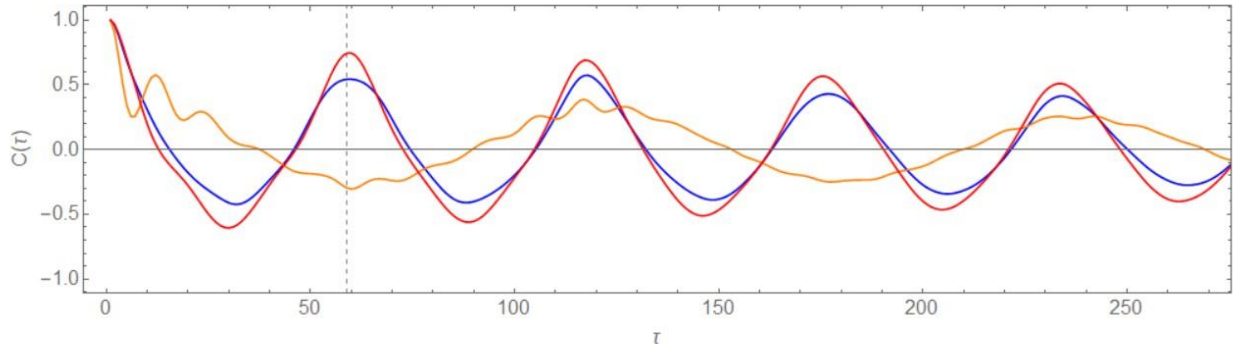

(d) Autocorrelation function of a selected frail older adult (F)

Figure S2. Autocorrelation functions calculated from the acceleration series shown in Figure S1. Panel (a) corresponds with a female young control subject of 24 yo, (b) a male control of 37 yo, (c) a female non-frail older adult of 74 yo, and (d) a male frail older adult of 81 yo. Every curve represents the autocorrelation function for every direction of movement: anterioposterior (blue curve), mediolateral (orange curve) and vertical (red curve). Vertical gridlines show the average step duration τ for each subject using as criteria the maximum value of vertical autocorrelation function [21].

ROC curve

We calculated a ROC curve for the gait speed parameter and found an Area Under the Curve of 0.83 m/s, which represents the gait speed threshold for the frail older adult. This curve had a sensitivity of 0.71, specificity of 0.92, precision of 0.81 and accuracy of 0.84.

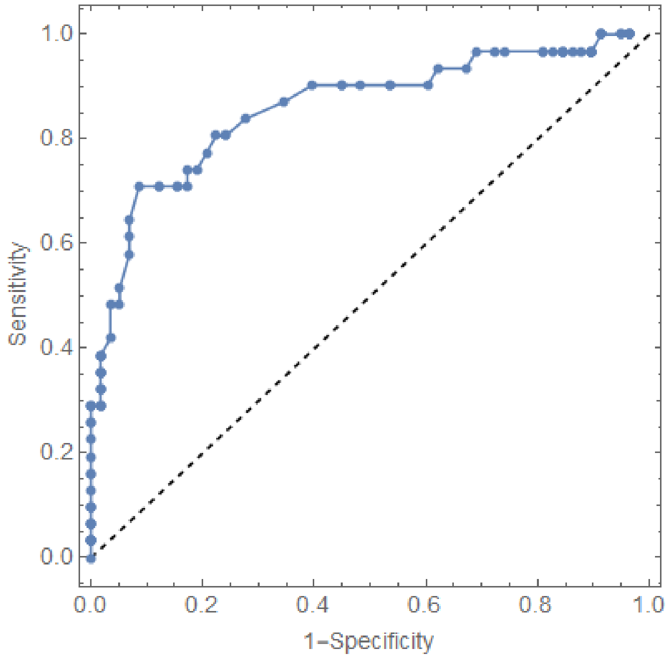

Figure S3. Representation of the ROC curve for the gait speed.

Standard deviation of triaxial accelerometry.

Table S1. Standard deviation of total acceleration SD (a), anteroposterior acceleration SD (a_{AP}), vertical acceleration SD (a_{VT}) and mediolateral acceleration SD (a_{ML}). Results are average values and standard errors for each population, young controls (C1), mature controls (C2), non-frail older adults (nF) and frail older adults (F).

**Variable**	**C1 (n=27)**	**C2 (n=16)**	**nF (n=15)**	**F (n=31)**	**p-value for all groups**
SD (a)	0.27 ± 0.01	0.29 ± 0.02	0.29 ± 0.02	0.21 ± 0.01	0.163
SD (a_{AP})	0.14 ± 0.05	0.16 ± 0.06	0.15 ± 0.04	0.11 ± 0.04	0.002
SD (a_{ML})	0.07 ± 0.02	0.08 ± 0.03	0.09 ± 0.02	0.07 ± 0.02	0.002
SD (a_{VT})	0.21 ± 0.06	0.22 ± 0.06	0.22 ± 0.04	0.16 ± 0.06	0.005

¶ $p < 0.05$ compared to C1 # $p < 0.05$ compared to C2 § $p < 0.05$ compared to nF
